# Emotional Dynamics in the Age of Misinformation

**DOI:** 10.1371/journal.pone.0138740

**Published:** 2015-09-30

**Authors:** Fabiana Zollo, Petra Kralj Novak, Michela Del Vicario, Alessandro Bessi, Igor Mozetič, Antonio Scala, Guido Caldarelli, Walter Quattrociocchi

**Affiliations:** 1 IMT Institute for Advanced Studies, Lucca, Italy; 2 Jožef Stefan Institute, Ljubljana, Slovenia 1000; 3 IUSS, Pavia, Italy; 4 ISC-CNR, Rome, Italy; University of Warwick, UNITED KINGDOM

## Abstract

According to the World Economic Forum, the diffusion of unsubstantiated rumors on online social media is one of the main threats for our society. The disintermediated paradigm of content production and consumption on online social media might foster the formation of homogeneous communities (echo-chambers) around specific worldviews. Such a scenario has been shown to be a vivid environment for the diffusion of false claim. Not rarely, viral phenomena trigger naive (and funny) social responses—e.g., the recent case of Jade Helm 15 where a simple military exercise turned out to be perceived as the beginning of the civil war in the US. In this work, we address the emotional dynamics of collective debates around distinct kinds of information—i.e., science and conspiracy news—and inside and across their respective polarized communities. We find that for both kinds of content the longer the discussion the more the negativity of the sentiment. We show that comments on conspiracy posts tend to be more negative than on science posts. However, the more the engagement of users, the more they tend to negative commenting (both on science and conspiracy). Finally, zooming in at the interaction among polarized communities, we find a general negative pattern. As the number of comments increases—i.e., the discussion becomes longer—the sentiment of the post is more and more negative.

## Introduction

People online get informed, discuss and shape their opinions [1–3]. Indeed, microblogging platforms such as Facebook and Twitter allow for the direct and disintermediated production and consumption of contents [4–7]. The information heterogeneity might facilitate users selective exposure to specific content and hence the aggregation in homophilous communities [8–15]. In such echo-chambers users interaction with different narratives is reduced and the resulting debates are often polarized (misinformation) [16–21].

Unfortunately, despite the enthusiastic rhetoric about *collective intelligence* [22–24], the direct and undifferentiated access to the knowledge production process is causing opposite effects—e.g., the recent case of Jade Helm 15 [25] where a simple military exercise turned out to be perceived as the beginning of the civil war in the US. Unsubstantiated rumors often jump the credulity barrier and trigger naive social responses. To an extent that, recently, the World Economic Forum labeled *massive digital misinformation* as one of the main threats to our society. Individuals may be uninformed or misinformed, and the debunking campaigns against unsubstantiated rumors do not seem to be effective [26].

Indeed, the factors behind the acceptance of a claim (whether substantiated or not) may be altered by normative social influence or by the coherence with the system of beliefs of the individual [27–31], making the preferential driver of contents the *confirmation bias*—i.e., the tendency to select and interpret information coherently with one’s system of beliefs.

In [16, 17, 19] it has been pointed out that the more users are exposed to unsubstantiated rumors, the more they are likely to jump the credulity barrier.

Recent studies [32, 33] pointed out that reading comments affects the perception of the topic and, thus, the discussion.

In this work we analyze a collection of *conspiracy* and *scientific* news sources in the Italian Facebook over a time span of four years. The main distinctive feature of the two categories of pages is the possibility to verify the reported content. Scientific news are generally fact-checked and are the results of a peer review process. Conversely, conspiracy news are generally partial information about a secret plot. We identify pages diffusing conspiracy news—i.e., pages promoting contents *neglected* by main stream media and scientific pages—aiming at diffusing scientific results. To have an exhaustive list of pages, we define the space of our investigation with the help of Facebook groups very active in debunking conspiracy stories and unverified rumors (*Protesi di Complotto*, *Che vuol dire reale*, *La menzogna diventa verità e passa alla storia*).

We target emotional dynamics inside and across content polarized communities. In particular, we apply sentiment analysis techniques to the comments of the Facebook posts, and study the aggregated sentiment with respect to scientific and conspiracy-like information. The sentiment analysis is based on a supervised machine learning approach, where we first annotate a substantial sample of comments, and then build a Support Vector Machine (SVM [34]) classification model. The model is then applied to associate each comment with one sentiment value: negative, neutral, or positive. The sentiment is intended to express the emotional attitude of Facebook users when posting comments.

Although other studies apply sentiment analysis to social media [35–38], our work is the first linking the interplay between communities emerging around shared narratives and specifically addressing the emotional dynamics with respect to misinformation spreading. Indeed, this work provides important insights toward the understanding of the social factors behind contents consumption and the formation of polarized and homophilous clusters with a specific interest in conspiracy-like information.

We focus on the emotional behavior of about 280k Facebook Italian users and through a thorough quantitative analysis, we find that the sentiment on conspiracy pages tends to be more negative than that on science pages. In addition, by focusing on polarized users—i.e., users mainly exposed to one specific content type (science or conspiracy)—we capture an overall increase of the negativity of the sentiment. According to our results, the more active polarized users are, the more they tend to be negative, both on science and conspiracy. Furthermore, the sentiment of polarized users is negative also when they interact with one another. Also, as the number of comments increases—i.e., the discussion turns longer– the sentiment is more and more negative.

## Results and Discussion

### Sentiment classification

Emotional attitude towards different topics can be roughly approximated by the sentiment expressed in texts. It is difficult to exactly formalize the sentiment measures since there are often disagreements between humans, and even individuals are not consistent with themselves.

In this study, as is often in the sentiment analysis literature [39], we have approximated the sentiment with an ordinal scale of three values: *negative* (−), *neutral* (0), and *positive* (+). Even with this rough approximation, and disagreements on single cases, it turns out that on a large scale, when one deals with thousands of sentiment assignments, the aggregated sentiment converges to stable values [40].

Our approach to automatic sentiment classification of texts is based on supervised machine learning. There are four steps: (i) a sample of texts is manually annotated with sentiment, (ii) the labeled set is used to train and tune a classifier, (iii) the classifier is evaluated on an independent test set or by cross-validation, and (iv) the classifier is applied to the whole set of texts.

We have collected over one million of Facebook comments. About 20k were randomly selected for manual annotation. We have engaged 22 native Italian speakers, active on Facebook, to manually annotate the comments by sentiment. The annotation is supported by a web-based platform Goldfinch—provided by Sowa Labs, http://www.sowalabs.com–and was accomplished in two months. About 20% of the comments were intentionally duplicated, in order to measure the mutual (dis)agreement of human annotators.

There are several measures to evaluate the inter-annotator agreement and performance of classification models. We argue that the inter-annotator agreement provides an upper bound that the best classification model can achieve. In practice, however, different learning algorithms have various limitations, and, most importantly, only a limited amount of training data is available. In order to compare the classifier performance to the inter-annotator agreement, we have selected three measures which are applied to evaluate both, performance and agreement: *Accuracy*, $\bar{F_{1}}$, and *Accuracy* ± 1. Exact definitions are in the Methods section, here we just briefly summarize them. *Accuracy* is the fraction of correctly classified examples for all three sentiment classes—no ordering between the classes is taken into account, and all three are treated equally. *F*_1_ is the harmonic mean of precision and recall for a selected class. $\bar{F_{1}}(-,+)$ is the average of *F*_1_ for the negative and positive class only, ignoring the neutral class. It is a standard measure of performance for sentiment classification [41]. The idea is that the misclassification of neutral sentiment can be ignored as it is less important then the extremes, i.e., negative or positive sentiment (however, it still affects their precision and recall). *Accuracy* ± 1 (an abbreviation for *Accuracy* *within 1*) completely ignores the neutral class. It counts as errors just the negative sentiment examples predicted as positive, and vice versa. It takes into account the fact that the neutral class is between the negative and the positive, and tolerates misclassifications within neighbouring classes.

Table 1 gives the evaluation results. In the case of the inter-annotator agreement, 3,262 examples were labeled twice by two different annotators, and measures assess their agreement. In the case of a sentiment classifier evaluation, we applied 10-fold cross-validation. The results in Table 1 are the average of 10 classifiers, with 95% confidence interval. One can see that the average classifier has reached a performance close to human agreement. In terms of extreme errors, i.e., 1 − *Accuracy* ± 1 the performance of the classifier is as good as the agreement between the annotators. However, in terms of *Accuracy* and $\bar{F_{1}}$, there is still some room for improvement. We speculate that the main reason for the gap is a relatively low number of annotated examples. Based on our experience in training SVM classifiers in other domains (such as stock market, elections, generic tweets, etc.), we estimate that about 50,000 to 100,000 training examples are needed to reach the level of the inter-annotator agreement.

**Table 1:** Comparison of the inter-annotator agreement and classifier performance over three evaluation measures. The results for an average sentiment classifier are from 10-fold cross-validation, with 95% confidence interval.

	Annotator agreement	Sentiment classifier
No. of testing examples	3,262	19,642
*Accuracy*(−, 0, +)	72.0%	64.8 ± 1.1%
$\bar{F_{1}}(-,+)$	73.3%	65.5 ± 1.0%
*Accuracy*±1(−, +)	97.2%	97.0 ± 0.3%

**Fig 1:**
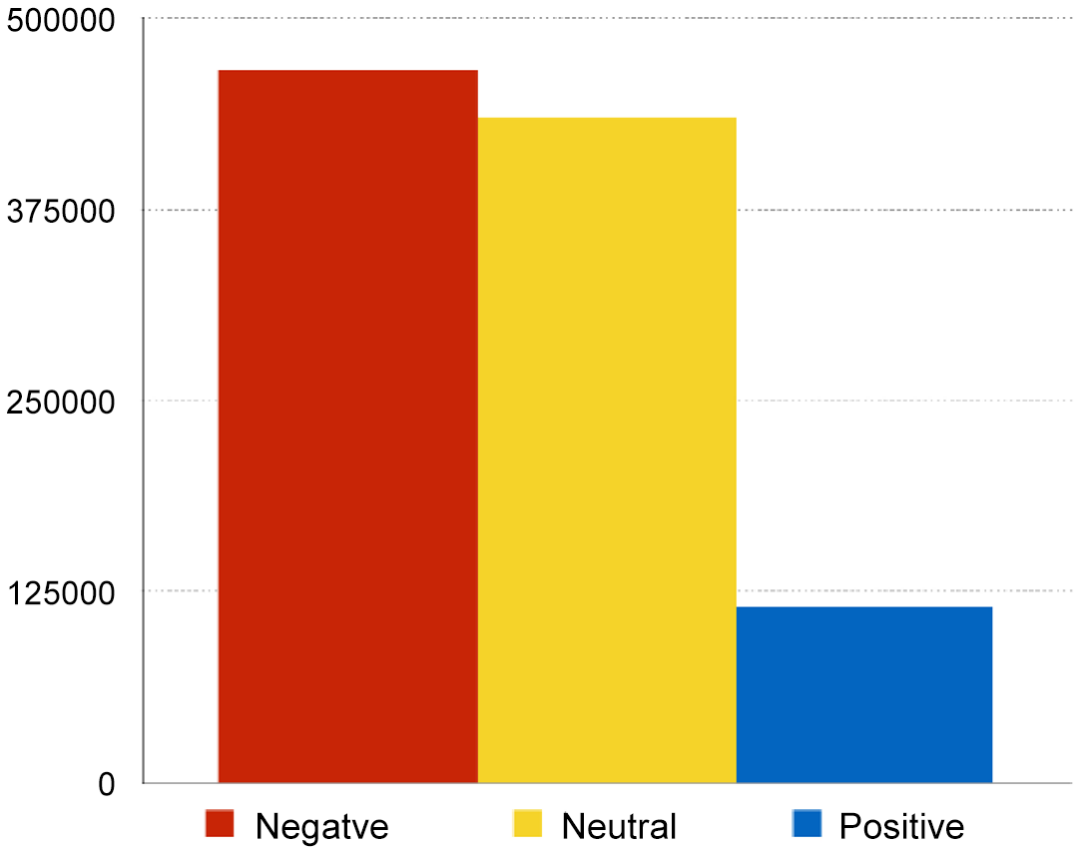
Sentiment distribution over the entire set of one million comments.

Fig 1 gives the distribution of sentiment values after applying the classification model to the entire set of over one million comments. We assume that the sentiment values are ordered, and that the difference from the neutral value to both extremes, negative and positive, is the same. Thus one can map the sentiment values from ordinal to a real-valued interval [−1, +1]. The mean sentiment over the entire set is −0.34, prevailingly negative.

### Sentiment on science and conspiracy posts

**Fig 2:**
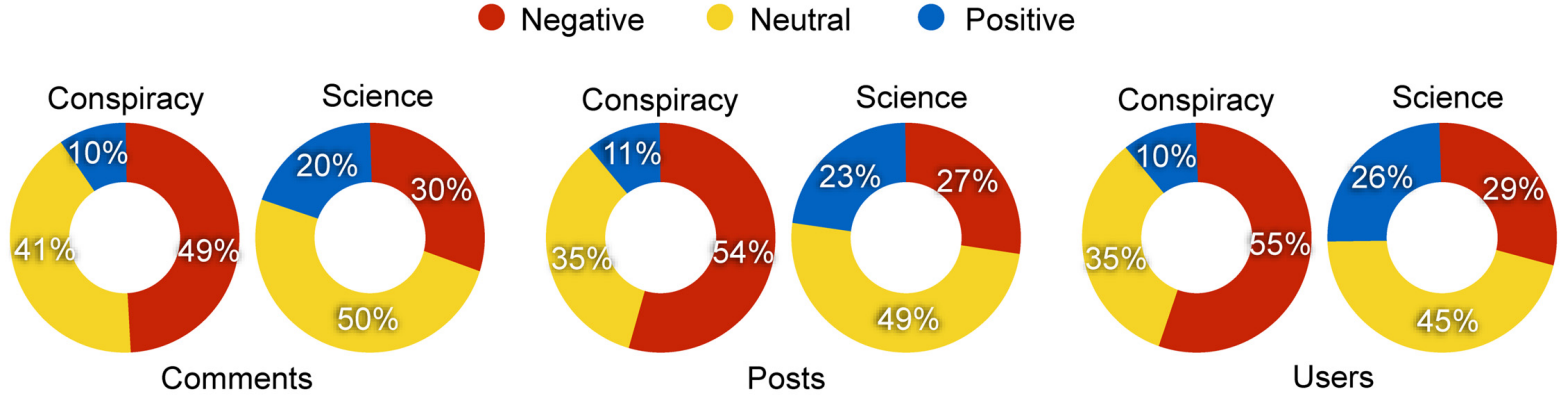
Sentiment on science and conspiracy pages. Proportions of negative, neutral and positive comments (*left*), posts (*center*), and users (*right*) both on science and conspiracy pages.

The sentiment analysis and classification task allowed us to associate each comment of our dataset to a sentiment value—i.e., −1 if *negative*, 0 if *neutral*, and 1 if *positive*. Taking all the comments of science and conspiracy posts, we can simply divide them into negative, neutral and positive (Fig 2, *left*), and analyze their proportions. We find that 70% of the comments on science pages is neutral or positive, differently from conspiracy pages (51%). Moreover, comments on science pages are twice as positive (20%) than those on conspiracy pages (10%).

To measure the effect induced on users by a post, we compute the average sentiment of all its comments. We grouped posts sentiment by defining three thresholds in order to equally divide the space; in particular, we say a post to be *negative* if the average sentiment ∈ [−1, −0.3], *neutral* if ∈ (−0.3,0.3), and *positive* if ∈ [0.3,1]. Fig 2 *(center)* shows the aggregated sentiment of science and conspiracy posts. Notice that the sentiment of conspiracy posts is mainly negative (54%), differently from science posts, for which the negative sentiment represents only the 27%. On the other hand, it is twice as positive for science posts (23%) than for conspiracy posts (11%).

When focusing on users, the approach is analogous. We define the sentiment of a user as the mean of the sentiment of all her comments. The mean sentiment for each user is then classified as negative, neutral, or positive by means of the same thresholds used for posts. Fig 2 *(right)* shows the aggregated sentiment both for science and conspiracy users. We find that the sentiment of users commenting on conspiracy pages is mainly negative (55%), while the sentiment of a small fraction of users (10%) is positive. On the contrary, the sentiment of users commenting on science pages is particularly neutral (45%), and negative only for 29% of users. Almost the same percentage (26%) is represented by positive sentiment.

### Sentiment and virality

**Fig 3:**
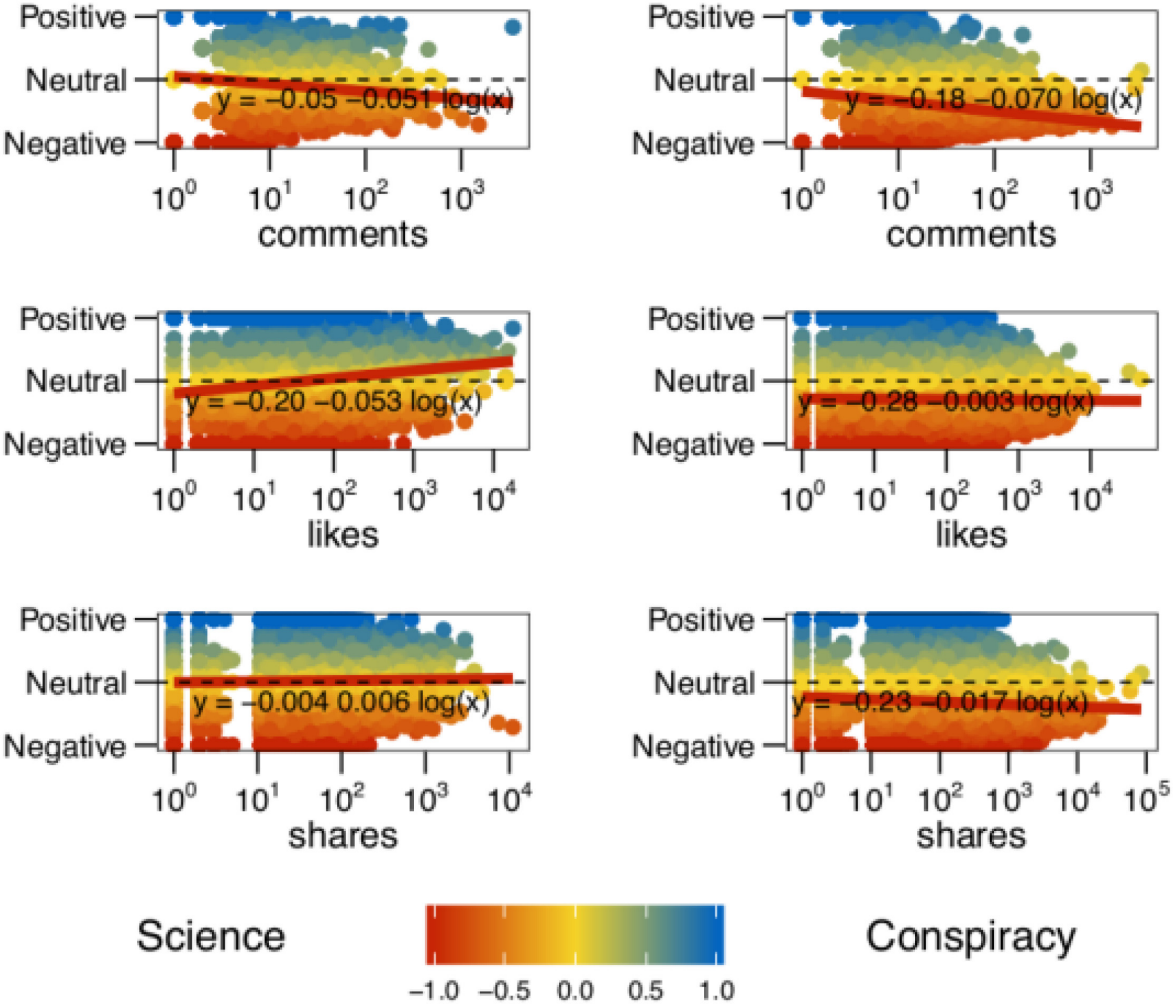
Sentiment and post consumption. Aggregated sentiment of posts as a function of their number of comments, likes, and shares, both for science (*left*) and conspiracy (*right*). Negative (respectively, neutral, positive) sentiment is denoted by red (respectively, yellow, blue) color. The sentiment has been regressed w.r.t. the logarithm of the number of comments/likes/shares.

Now we focus on the interplay between the virality of a post and its generated sentiment. In particular we want to understand how the sentiment varies for increasing levels of comments, likes, and shares. Notice that each of these actions has a particular meaning [42–44]. A *like* stands for a positive feedback to the post; a *share* expresses the will to increase the visibility of a given information; and a *comment* is the way in which online collective debates take form around the topic promoted by posts. Comments may contain negative or positive feedbacks with respect to the post. Fig 3 shows the aggregated sentiment of a post as a function of its number of comments *(top)*, likes *(center)*, and shares *(bottom)* both for science *(left)* and conspiracy *(right)* posts. The sentiment has been regressed w.r.t. the logarithm of the number of comments (resp., likes, shares). We do not show confidence intervals, since they are defined (C.I. 95%) as $\bar{X}\pm S.E.=\bar{X}\pm 1.96\frac{\sigma }{\sqrt{n}}$ and when *n* → ∞, *S*.*E*. = 0. We notice that the sentiment decreases both for science and conspiracy when the number of comments of the post increases. However, we also note that it becomes more positive for science posts when the number of likes and shares increase, differently from conspiracy posts.

To assess the direct relationship between the number of comments and the negativity of the sentiment, a randomization test was performed. In particular, we took all the comments of science (resp., conspiracy) posts and randomly reassigned the original sentiments. Then, we regressed the sentiment w.r.t. the number of comments and compared the obtained slope with the one shown in Fig 3 *(top)*. Over 10k randomized tests, the obtained slope was always greater than the original one. More precisely, while the slope for the original comments for Science is equal to −0.051 (resp., −0.070 for Conspiracy), the quantiles of the distribution of the slopes in the randomized test are: *Q*_0_ = −0.010, *Q*_1_ = −0.002, *Q*_2_ = −0.00002, *Q*_3_ = 0.002, *Q*_4_ = 0.010 (resp., *Q*_0_ = −0.004, *Q*_1_ = −0.0008, *Q*_2_ = −0.000004, *Q*_3_ = 0.0008, *Q*_4_ = 0.005, for Conspiracy). Therefore, given that the negative relationship between the sentiment and the length of the discussion disappears when the comment sentiments are randomized, we conclude that the length of the discussion is a relevant dimension when considering the negativity of the sentiment.

Summarizing, we found that both comments and posts, as well as users of conspiracy pages tend to be much more negative than those of science pages. Interestingly, the sentiment becomes more and more negative when the number of comments of the post increases—i.e., the discussion becomes longer– both on science and conspiracy pages. However, differently from conspiracy posts, when the number of likes and shares increases, the aggregated sentiment of science posts becomes more and more positive.

### Sentiment and users activity

In this section we aim at understanding more in depth how the sentiment changes with respect to users’ engagement in one of the two communities. Previous works [17, 19, 20] showed that the distribution of the users activity on the different contents is highly polarized. Therefore we now want to focus on the sentiment of polarized users. More precisely, we say a user to be polarized on science (respectively, on conspiracy) if she left more than 95% of her likes on science (respectively, on conspiracy) posts (for further details about the effect of the thresholding refer to the Methods Section).

**Fig 4:**
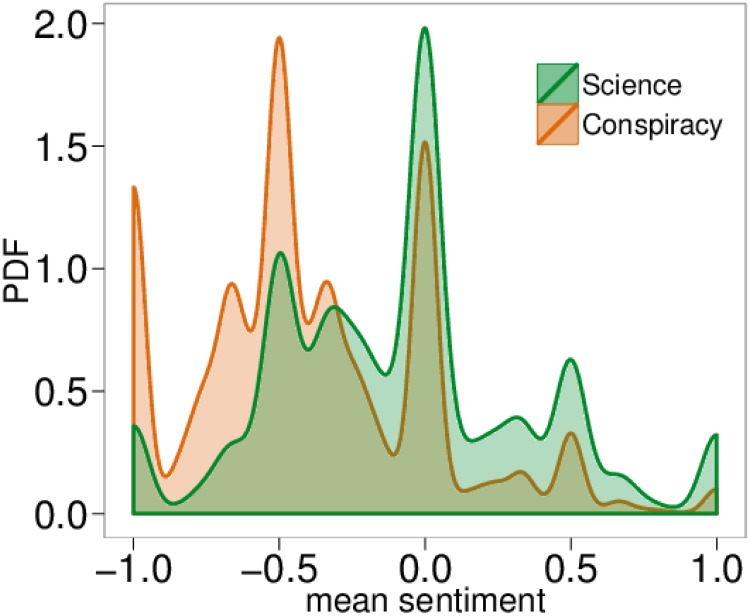
Sentiment and polarization. Probability Density Function (PDF) of the mean sentiment of polarized users having commented at least twice, where −1 corresponds to negative sentiment, 0 to neutral and 1 to positive.

Therefore, we take all polarized users having commented at least twice, i.e., 14,887 out of 33,268 users polarized on science and 67,271 out of 135,427 users polarized on conspiracy. Fig 4 shows the Probability Density Function (PDF) of the mean sentiment of polarized users with at least two comments. In Table 2 we compare the mean sentiment of all users and polarized users having commented at least twice. Our results show that the overall negativity increases w.r.t. all users, such a feature is more evident on the conspiracy side.

**Table 2:** Sentiment and polarized users.

	Science		Conspiracy	
Sentiment	All users	Polarized	All users	Polarized
*Negative*	29%	34%	55%	66%
*Neutral*	45%	46%	35%	27%
*Positive*	26%	20%	10%	7%

Mean sentiment of all users and polarized users having commented at least twice.

**Fig 5:**
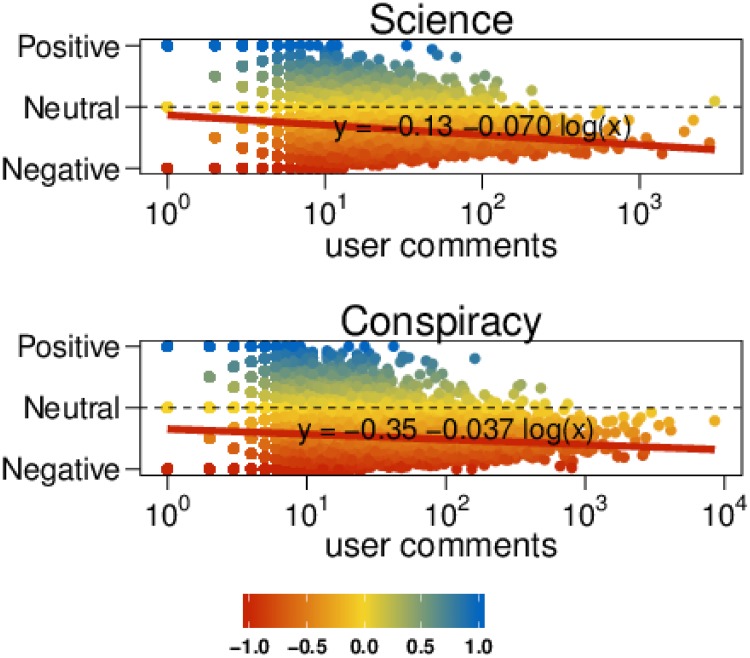
Sentiment and commenting activity. Average sentiment of polarized users as a function of their number of comments. Negative (respectively, neutral, positive) sentiment is denoted by red (respectively, yellow, blue) color. The sentiment has been regressed w.r.t. the logarithm of the number of comments.

We now want to investigate how the mean sentiment of a user changes with respect to her commenting activity –i.e., when her total number of comments increases. In Fig 5 we show the mean sentiment of polarized users as a function of their number of comments. The more active a polarized user is, the more she tends toward negative values both on science and conspiracy posts. The sentiment has been regressed w.r.t. the logarithm of the number of comments. Interestingly, the sentiment of science users decreases faster than that of conspiracy users. We performed a randomization test taking all comments on both categories and then randomly reassigning the original sentiments. Then, we regressed the sentiment w.r.t. the number of comments and compared the obtained slope with the one shown in Fig 5. The obtained slope over 10k randomized tests was always greater than the original one. In particular, while the slope for the original comments for Science is equal to −0.070 (resp., −0.037 for Conspiracy), the quantiles of the distribution of the slopes in the randomized test are: *Q*_0_ = −0.006, *Q*_1_ = −0.001, *Q*_2_ = 0.00001, *Q*_3_ = 0.001, *Q*_4_ = 0.006 (resp., *Q*_0_ = −0.003, *Q*_1_ = −0.0005, *Q*_2_ = 0.00001, *Q*_3_ = 0.0005, *Q*_4_ = 0.003, for Conspiracy). Therefore users activity is a relevant dimension when considering the value of the sentiment, which is more and more negative on both categories when the users activity increases.

### Interaction across communities

In this section we aim at investigating the sentiment when usual consumers of science and conspiracy news meet. To do this we pick all posts representing the arena where the debate between science and conspiracy users takes place. In particular, we select all posts commented at least once by both a user polarized on science and a user polarized on conspiracy. We find 7,751 such posts (out of 315,567) –reinforcing the fact that the two communities of users are strictly separated and do not often interact with one another.

**Fig 6:**
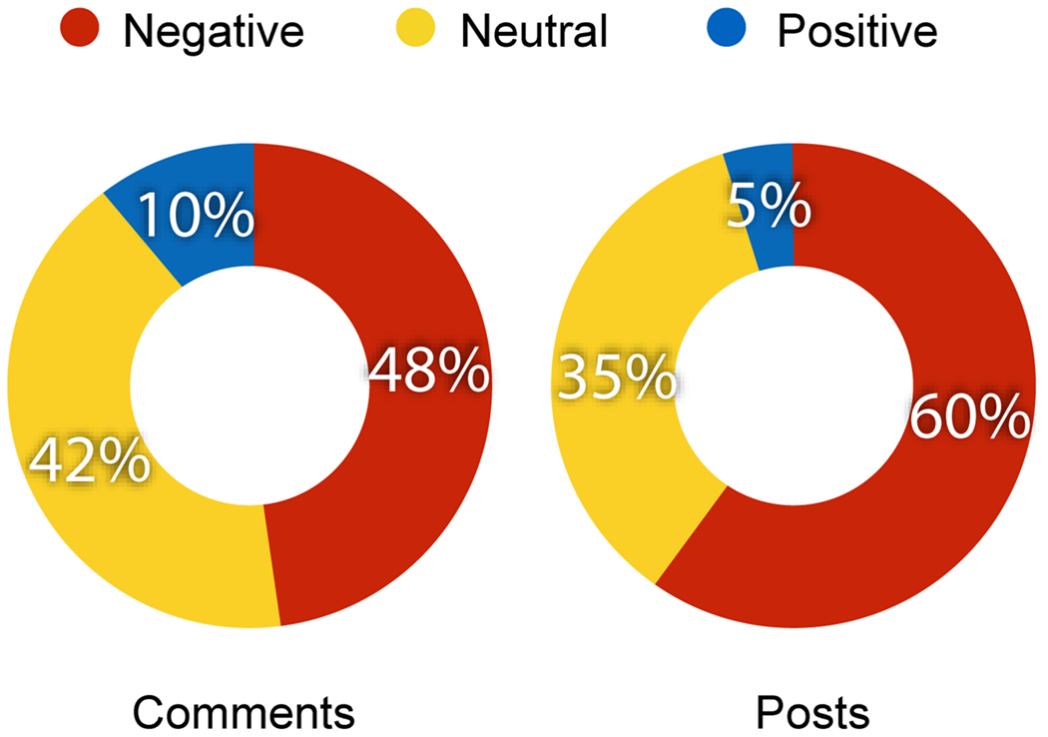
Sentiment between communities. Proportions of negative, neutral, and positive comments (*left*) and posts (*right*) of all the posts commented at least once by both a user polarized on science and a user polarized on conspiracy.

**Fig 7:**
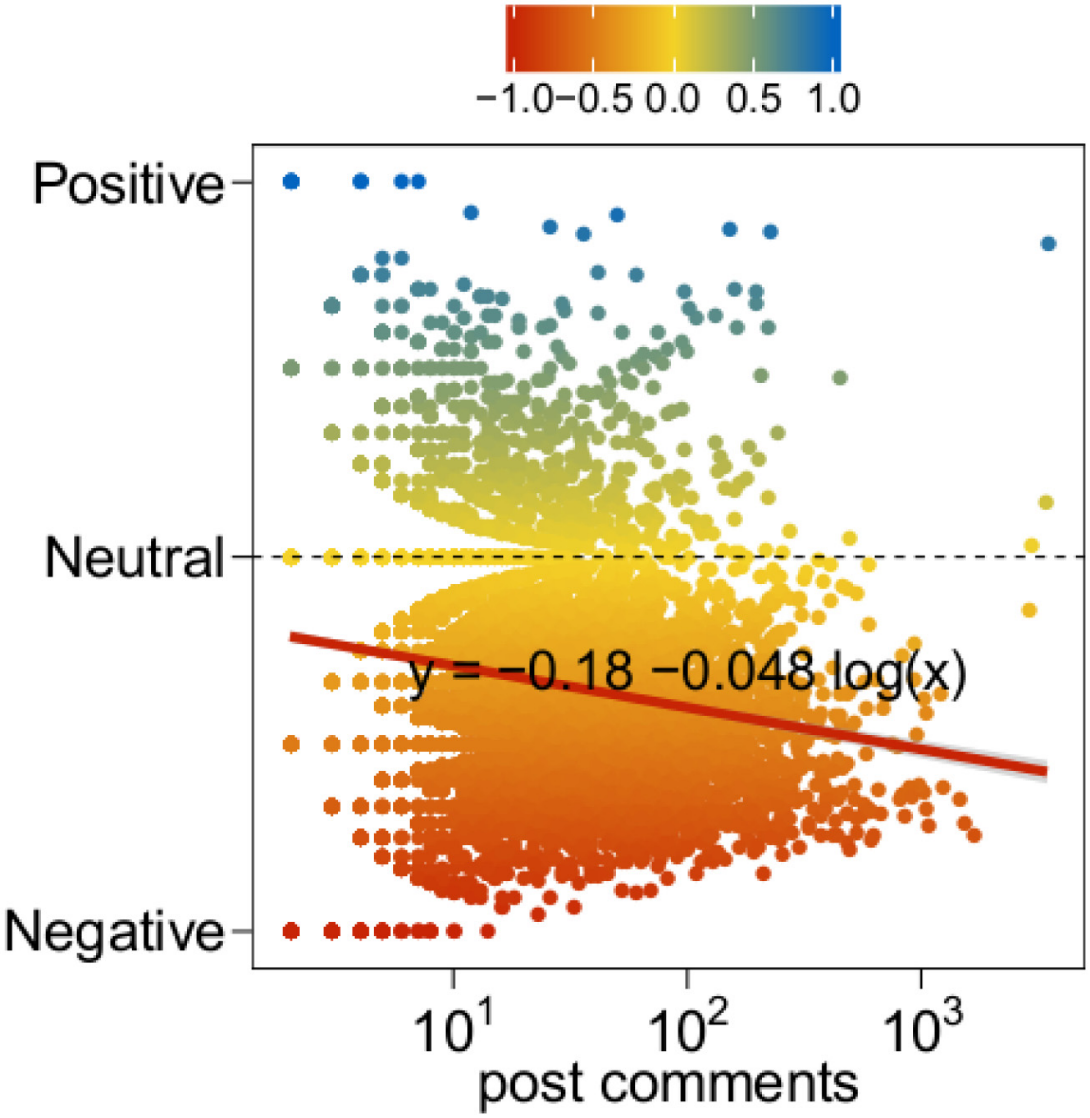
Sentiment and discussion. Aggregated sentiment of posts as a function of their number of comments. Negative (respectively, neutral, positive) sentiment is denoted by red (respectively, yellow, blue) color.

In Fig 6 we show the proportions of negative, neutral, and positive comments (*left*) and posts (*right*). The aggregated sentiment of such posts is slightly more negative (60%) than for general posts (54% for conspiracy, 27% for science, see Fig 2). When focusing on comments, we have similar percentages of neutral (42%) and negative (48%) comments, while a small part (10%) is represented by positive comments. We want to understand if the sentiment correlates with the length of the discussion. Hence, we analyze how the sentiment changes when the number of comments of the post increases, as we previously did for *general* posts (Fig 3). Fig 7 shows the aggregated sentiment of such posts as a function of their number of comments. Clearly, as the number of comments increases –i.e., the discussion becomes longer– the sentiment is more and more negative. Moreover, comparing with Fig 3, when communities interact with one another, posts show a higher concentration of negative sentiment.

Also in this case we performed a randomization test taking all the comments and randomly reassigning the original sentiments. Then, we regressed the sentiment w.r.t. the number of comments and compared the obtained slope with the one shown in Fig 7. Over 10k randomized tests, the obtained slope was always greater than the original one. In particular, while the slope for the original comments is equal to −0.048, the quantiles of the distribution of the slopes in the randomized test are: *Q*_0_ = −0.009, *Q*_1_ = −0.002, *Q*_2_ = 0.00004, *Q*_3_ = 0.002, *Q*_4_ = 0.009. Therefore, we conclude that the length of the discussion does affect the negativity of the sentiment.

## Conclusions

In this work we analyzed the emotional dynamics on pages of opposite worldviews, science and conspiracy. Previous works [17, 19, 20] showed that users are strongly polarized towards the two narratives. Moreover, we found that users of both categories seem to not distinguish between verified contents and unintentional false claims. In this manuscript we focused on the emotional behavior of the same users on Facebook. In general, we noticed that the sentiment on conspiracy pages tends to be more negative than that on science pages. In addition, by focusing on polarized users, we identified an overall increase of the negativity of the sentiment. In particular, the more active polarized users, the more they tend to be negative, both on science and conspiracy. Furthermore, the sentiment of polarized users is negative also when they interact with one another. Also in this case, as the number of comments increases –i.e., the discussion becomes longer– the sentiment of the post is more and more negative. This work provides important insights about the emotional dynamics in a disintermediated environment. Indeed, recent studies [32, 33] pointed out that reading comments of other user may affect the discussion. Our findings confirm such a phenomenon and make explicit that the longer the discussion the more negative the sentiment. In particular, discussions around conspiracy news degenerate faster than the scientific one. This latter point opens to interesting question about the quasi-religious mentality of conspiracists [45] and the way in which such an echo-chamber digests and debate news and events.

## Methods

### Ethics statement

The entire data collection process has been carried out exclusively through the Facebook Graph API [46], which is publicly available, and for the analysis (according to the specification settings of the API) we used only public available data (users with privacy restrictions are not included in the dataset). The pages from which we download data are public Facebook entities (can be accessed by anyone). User content contributing to such pages is also public unless the user’s privacy settings specify otherwise and in that case it is not available to us.

### Data collection

We identified two main categories of pages: conspiracy news –i.e. pages promoting contents neglected by main stream media– and science news. The first category includes all pages diffusing conspiracy information –pages which disseminate controversial information, most often lacking supporting evidence and sometimes contradictory to the official news (i.e., conspiracy theories). The second category is that of scientific dissemination, including scientific institutions and scientific press having the main mission to diffuse scientific knowledge. Note that we do not focus on the truth value of information but rather on the possibility of verifying the content of the page. While the latter is an easy task for scientific news—e.g., by identifying the authors of the study or if the paper passed a peer review process—it usually becomes more difficult for conspiracy-like information, if not unfeasible. We defined the space of our investigation with the help of Facebook groups very active in debunking conspiracy theses (*Protesi di Complotto*, *Che vuol dire reale*, *La menzogna diventa verità e passa alla storia*). We categorized pages according to their contents and their self description. The resulting dataset –downloaded over a timespan of four years (2010 to 2014)– is composed of 73 public Italian Facebook pages and it is the same used in [19] and [20]. To the best of our knowledge, the final dataset is the complete set of all scientific and conspiracy information sources active in the Italian Facebook scenario. Table 3 summarizes the details of our data collection.

**Table 3:** Breakdown of the Facebook dataset.

	Total	Science	Conspiracy
Pages	73	34	39
Posts	270,666	62,075	208,591
Likes	9,164,781	2,505,399	6,659,382
Comments	1,017,509	180,918	836,591
Shares	17,797,819	1,471,088	16,326,731
Likers	1,196,404	332,357	864,047
Commenters	279,972	53,438	226,534

### Classification and annotator agreement measures

Our approach to sentiment classification of texts is based on supervised machine learning, where a sample of texts is first manually annotated with sentiment and then used to train and evaluate a classifier. The classifier is then applied to the whole corpus. The measures to assess the agreement between annotators and the quality of the classifier are based on coincidence and confusion matrices, respectively.

Annotators were asked to label each text with *negative* ≺ *neutral* ≺ *positive* sentiment. When two annotators are given the same text, they can either agree (both give the same label) or disagree (they give different labels). The annotators can disagree in two ways: one label is *neutral* while the other is extreme (*negative* or *positive*), or both are extreme: one *negative* and one *positive* —we call this severe disagreement. A convenient way to represent the overall (dis)agreement between the annotators is a coincidence matrix, where each text that is annotated twice appears in the table twice. Table 4 gives a generic 3 × 3 annotator agreement table, while the actual data are in Tables 5 and 6. All agreements are on the diagonal of the table. As the labels are ordered (*negative* ≺ *neutral* ≺ *positive*), the further the cell from the diagonal, the more severe is the error. From such a table one can calculate the annotator agreement (the sum of the main diagonal divided by the number of all the elements in the table) and the severe disagreement: the sum of top right and bottom left corners divided by the number of all the elements in the table.

**Table 4:** A generic 3 × 3 coincidence matrix/confusion matrix. An element 〈*x*, *y*〉 denotes the number of examples from the actual class *x*, predicted as class *y*.

Actual/Predicted	*Negative*	*Neutral*	*Positive*	Total
*Negative*	〈−, −〉	〈−,0〉	〈−, +〉	〈−,*〉
*Neutral*	〈0, −〉	〈0,0〉	〈0, +〉	〈0,*〉
*Positive*	〈+, −〉	〈+,0〉	〈+, +〉	〈+,*〉
**Total**	〈*, −〉	〈*,0〉	〈*, +〉	*N*

**Table 5:** A coincidence matrix for the inter-annotator agreement, excluding self-agreement.

	*Negative*	*Neutral*	*Positive*	Total
*Negative*	2,482	545	90	3,117
*Neutral*	545	1,474	277	2,296
*Positive*	90	277	744	1,111
**Total**	3,117	2,296	1,111	6,524

**Table 6:** A coincidence matrix for the annotators’ self-agreement.

	*Negative*	*Neutral*	*Positive*	Total
*Negative*	486	57	6	549
*Neutral*	57	434	19	510
*Positive*	6	19	196	221
**Total**	549	510	221	1280

To compare the predictions of a classifier to a golden standard (manually annotated data, in our case), a confusion matrix is used. Table 4 also represents a generic 3 × 3 confusion matrix for the (ordered) sentiment classification case. Each element 〈*x*, *y*〉 represents the number of examples from the actual class *x*, predicted as class *y*. All agreements/correct predictions are in the diagonal of the table. In the ordinal classification case, the further the cell from the diagonal, the more severe is the error.

*Accuracy* is the fraction of correctly classified examples:

$$\begin{array}{c} Accuracy=\frac{\langle -,-\rangle +\langle 0,0\rangle +\langle +,+\rangle }{N} \end{array}$$

*Accuracy within n* [47] allows for a wider range of predictions to be considered correct. We use *Accuracy within 1* (*Accuracy*±1) where only misclassifications from *negative* to *positive* and vice-versa are considered incorrect:

$$\begin{array}{c} Accuracy\;\pm \;1\left(-,+\right)=1-\frac{\langle +,-\rangle +\langle -,+\rangle }{N} \end{array}$$

$\bar{F_{1}}(+,-)$ is the macro-averaged *F*-score of the positive and negative classes, a standard evaluation measure [41] used also in the SemEval competition (http://alt.qcri.org/semeval2015/) for sentiment classification tasks:

$$\begin{array}{c} \bar{F_{1}}\left(+,-\right)=\frac{F_{1+}+F_{1-}}{2} \end{array}$$

*F*_1_ is the harmonic mean of *Precision* and *Recall* for each class [48]:

$$\begin{array}{c} F_{1}=2\cdot \frac{Precision\cdot Recall}{Precision+Recall} \end{array}$$

*Precision* for class *x* is the fraction of correctly predicted examples out of all the predictions with class *x*:

$$\begin{array}{c} Precision_{x}=\frac{\langle x,x\rangle }{\langle *,x\rangle } \end{array}$$

*Recall* for class *x* is the fraction of correctly predicted examples out of all the examples with actual class *x*:

$$\begin{array}{c} Recall_{x}=\frac{\langle x,x\rangle }{\langle x,*\rangle } \end{array}$$

From the above tables and definitions, one can see that the annotator agreement is equivalent to *Accuracy* and that severe disagreement is equivalent to 1 − *Accuracy*±1. $\bar{F_{1}}$ has no counterpart between the annotator agreement measures, but is a standard measure in evaluation of sentiment classifiers. On the other hand, Cohen’s kappa [49] is a standard measure of inter-rater agreement, but rarely used to evaluate classification models. The original Cohen’s kappa is applicable to categorical (unordered) classes, and weighted kappa was devised for ordered classes. We use *Cohen’s weighted kappa* [50] to compare the inter-annotator agreement and self-agreement.

### Data annotation

Data annotation is a process in which some predefined labels are assigned to each data point. A subset of 19,642 comments from the Facebook dataset of one million (Table 3) was selected for manual sentiment annotation and later used to train a sentiment classifier. A user-friendly web and mobile devices annotation platform Goldfinch—provided by Sowa Labs, http://www.sowalabs.com/–was used.

Trustworthy Italian native speakers, active on Facebook, were engaged for the annotations. The annotation task was to label each Facebook comment—isolated from its context—as *negative*, *neutral*, or *positive*. The guideline given to the annotators was to estimate the emotional attitude of the user when posting a comment to Facebook. The exact question an annotator should answer was: ‘Is the user happy (pleased, satisfied), or unhappy (angry, sad, frustrated), or neutral?’ A dedicated Facebook group was formed to dispatch detailed annotation instructions, to provide a forum for discussion, and to post ongoing annotation results which stimulated the annotators to contribute. During the annotation process, which lasted for about two months, the annotator performance was monitored in terms of the inter-annotator agreement and self-agreement, based on 20% of the comments which were intentionally duplicated. No compensation, other then gratitude and personal satisfaction for contributing to interesting scientific research, was awarded.

The annotation process resulted in 19,642 sentiment labeled comments, 3,902 of them annotated twice. Out of 3,902 duplicates, 3,262 were polled twice to two different annotators and are used to assess the inter-annotator agreement, and 640 were polled twice to the same annotator and are used to asses the annotators’ self-agreement. The coincidence matrices with the inter-annotator agreement and self-agreement are in Tables 5 and 6, respectively.

Note that, in a coincidence matrix, each annotated example appears twice (once for each of the two annotators), thus the matrix is symmetric. This is in contrast to a confusion matrix where one knows the ground truth, and the matrix values are the number of examples in the actual and predicted classes.

The four evaluation measures, defined above, were used to quantify the inter-annotator and the annotators’ self-agreement. The results are in Table 7.

**Table 7:** Comparison of the inter-annotator and self-agreement over four evaluation measures.

	Inter-annotator agreement	Annotators’ self-agreement
No. of overlapping examples	3,262	640
*Accuracy*(−,0, +)	72.0%	87.2%
$\bar{F_{1}}(-,+)$	73.3%	88.7%
*Accuracy*±1(−, +)	97.2%	99.1%
*Cohen’s weighted kappa*	0.61	0.82

### Classification

Ordinal classification, also known as ordinal regression, is a form of multi-class classification where there is a natural ordering between the classes, but no meaningful numeric difference between them [47]. We treat sentiment classification as an ordinal regression task with three ordered classes. We apply the wrapper approach, described in [51], with two linear-kernel Support Vector Machine (SVM) [34] classifiers. SVM is a state-of-the-art supervised learning algorithm, well suited for large scale text categorization tasks, and robust on large feature spaces. The two SVM classifiers were trained to distinguish the extreme classes (*negative* and *positive*) from the rest (*neutral* plus *positive*, and *neutral* plus *negative*, respectively). During prediction, if both classifiers agree, they yield the common class, otherwise, if they disagree, the assigned class is *neutral*.

The sentiment classifier was trained and tuned on the training set of 15,714 annotated comments. The comments were processed into the standard Bag-of-Words (BoW) representation, with the following settings: lemmatized BoW include unigrams and bigrams, minimum n-gram frequency is five, TF-IDF weighting, no stop-word removal, and normalized vectors. Additional features and settings were chosen, based on the results of 10-fold stratified cross-validation on the training set: normalization of diacritical characters, url replacement, length of text, presence of upper cased words, negation (language specific), swearing (language specific), positive words from a predefined dictionary (language specific), unusual punctuation (several exclamation or question marks, …), unusually repeated characters, happy or sad emoticons in the text, and their presence at the end of the sentence.

The trained sentiment classifier was then evaluated on a disjoint test set of the remaining 3,928 comments. The confusion matrix between the annotators (actual classes) and the classifier (predicted classes) is in Table 8. The sentiment class distribution, after applying the classifier to the whole set of one million Facebook comments, is in Fig 1.

**Table 8:** A confusion matrix of the sentiment classifier on the test set.

Actual/Predicted	*Negative*	*Neutral*	*Positive*	Total
*Negative*	1,208	501	32	1,741
*Neutral*	509	987	103	1,599
*Positive*	86	183	319	588
**Total**	1,803	1,671	454	3,928

Another evaluation was performed by a 10-fold cross-validation on the complete set of 19,642 training examples. The confusion matrix between the annotators and the 10 classifiers is in Table 9. The averaged evaluation measures over 10 classifiers, with 95% confidence interval are in Table 1.

**Table 9:** A confusion matrix of sentiment classifiers on the 10-fold cross-validated complete training set.

Actual/Predicted	*Negative*	*Neutral*	*Positive*	Total
*Negative*	5,779	2,669	302	8,750
*Neutral*	1,969	5,090	839	7,898
*Positive*	293	834	1,867	2,994
**Total**	8,041	8,593	3,008	19,642

### Statistical tools

To characterize random variables, a main tool is the probability distribution function (PDF), which gives the probability that a random variable *X* assumes a value in the interval [*a*, *b*], i.e. $P(a\leq X\leq b)=\int_{a}^{b}f(x)dx$.

#### Labeling algorithm

**Fig 8:**
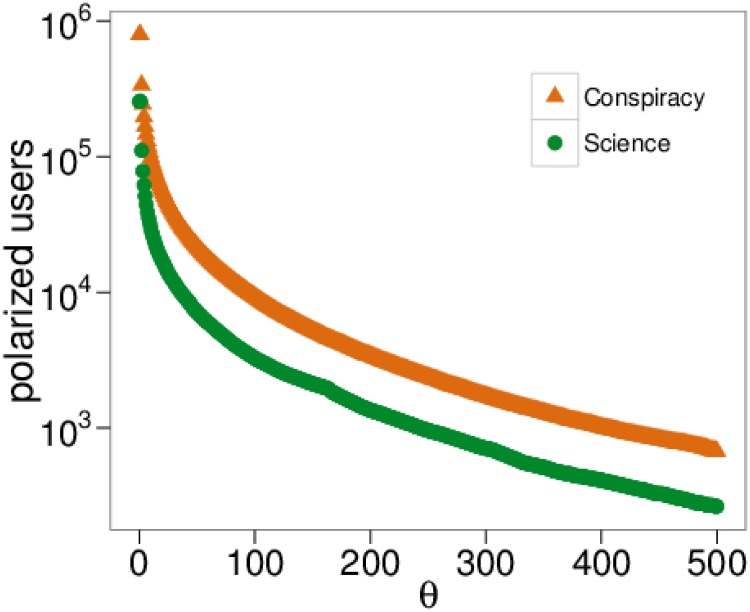
Polarized users and activity. The number of polarized users as a function of the thresholding value *θ* on the two categories.

**Fig 9:**
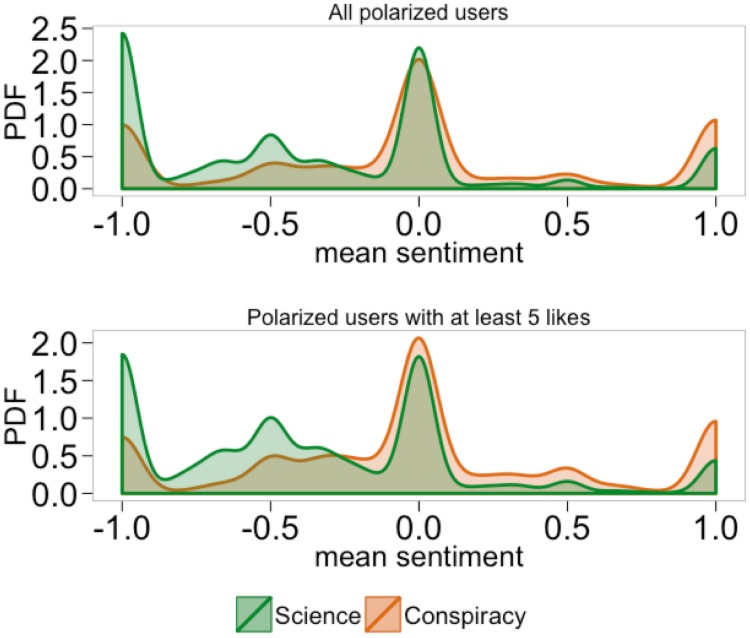
Sentiment of Polarized Users. Probability Density Function (PDF) of the mean sentiment of all polarized users (top) and polarized users with at least five likes, where −1 corresponds to negative sentiment, 0 to neutral and 1 to positive.

**Fig 10:**
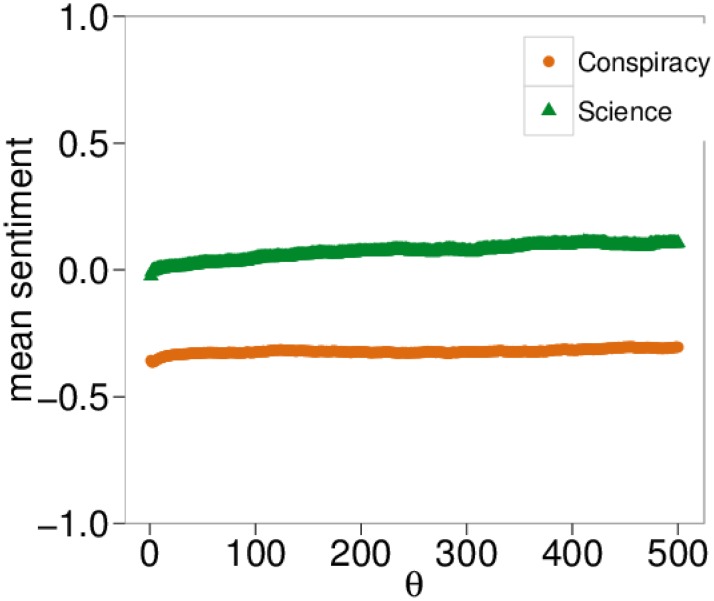
Sentiment and Engagement. Average sentiment of polarized users as a function of the threshold *θ*, i.e., the engagement degree, intended as the number of likes a polarized user put in her own category.

The labeling algorithm may be described as a thresholding strategy on the total number of users likes. Considering the total number of likes of a user *L*_*u*_ on both posts *P* in categories *S* and *C*. Let *l*_*s*_ and *l*_*c*_ define the number of likes of a user *u* on *P*_*s*_ or *P*_*c*_, respectively denoting posts from scientific or conspiracy pages. Then, the total like activity of a user on one category is given by $\frac{l_{s}}{L_{u}}$. Fixing a threshold *θ* we can discriminate users with enough activity on one category. More precisely, the condition for a user to be labeled as a polarized user in one category can be described as $\frac{l_{s}}{L_{u}}$ ∨ $\frac{l_{c}}{L_{u}}$ > *θ*. In Fig 8 we show the number of polarized users as a function of *θ*. Both curves decrease with a comparable rate. Fig 9 shows the Probability Density Function (PDF) of the mean sentiment of all polarized users *(top)* and polarized users with at least five likes *(bottom)*. Note that both densities are qualitatively similar. In Fig 10 we show the mean sentiment of polarized users as a function of the threshold *θ*.

### List of pages

In this section, we provide the full list of Facebook pages of our dataset. Table 10 lists scientific pages, while Table 11 lists conspiracy pages.

**Table 10:** Scientific news sources. List of Facebook pages diffusing main stream scientific news and their url.

	Page Name	Link
1	Scientificast.it	www.facebook.com/129133110517884
2	CICAP	www.facebook.com/32775139194
3	OggiScienza	www.facebook.com/106965734432
4	Query	www.facebook.com/128523133833337
5	Gravità Zero	www.facebook.com/138484279514358
6	COELUM Astronomia	www.facebook.com/81631306737
7	MedBunker	www.facebook.com/246240278737917
8	In Difesa della Sperimentazione Animale	www.facebook.com/365212740272738
9	Italia Unita per la Scienza	www.facebook.com/492924810790346
10	Scienza Live	www.facebook.com/227175397415634
11	La scienza come non l’avete mai vista	www.facebook.com/230542647135219
12	LIBERASCIENZA	www.facebook.com/301266998787
13	Scienze Naturali	www.facebook.com/134760945225
14	Perché vaccino	www.facebook.com/338627506257240
15	Le Scienze	www.facebook.com/146489812096483
16	Vera scienza	www.facebook.com/389493082245
17	Scienza in rete	www.facebook.com/84645527341
18	Galileo, giornale di scienza e problemi globali	www.facebook.com/94897729756
19	Scie Chimiche: Informazione Corretta	www.facebook.com/351626174626
20	Complottismo? No grazie	www.facebook.com/399888818975
21	INFN—Istituto Nazionale di Fisica Nucleare	www.facebook.com/45086217578
22	Signoraggio: informazione corretta	www.facebook.com/279217954594
23	JFK informazione corretta	www.facebook.com/113204388784459
24	Scetticamente	www.facebook.com/146529622080908
25	Vivisezione e Sperimentazione Animale, verità e menzogne	www.facebook.com/548684548518541
26	Medici Senza Frontiere	www.facebook.com/65737832194
27	Task Force Pandora	www.facebook.com/273189619499850
28	VaccinarSI	www.facebook.com/148150648573922
29	Lega Nerd	www.facebook.com/165086498710
30	Super Quark	www.facebook.com/47601641660
31	Curiosità Scientifiche	www.facebook.com/595492993822831
32	Minerva—Associazione di Divulgazione Scientifica	www.facebook.com/161460900714958
33	Pro-Test Italia	www.facebook.com/221292424664911
34	Uniti per la Ricerca	www.facebook.com/132734716745038

**Table 11:** Conspiracy news sources. List of Facebook pages diffusing conspiracy news and their url.

	**Page Name**	**Link**
1	Scienza di Confine	www.facebook.com/188189217954979
2	CSSC—Cieli Senza Scie Chimiche	www.facebook.com/253520844711659
3	STOP ALLE SCIE CHIMICHE	www.facebook.com/199277020680
4	Vaccini Basta	www.facebook.com/233426770069342
5	Tanker Enemy	www.facebook.com/444154468988487
6	SCIE CHIMICHE	www.facebook.com/68091825232
7	MES Dittatore Europeo	www.facebook.com/194120424046954
8	Lo sai	www.facebook.com/126393880733870
9	AmbienteBio	www.facebook.com/109383485816534
10	Eco(R)esistenza	www.facebook.com/203737476337348
11	curarsialnaturale	www.facebook.com/159590407439801
12	La Resistenza	www.facebook.com/256612957830788
13	Radical Bio	www.facebook.com/124489267724876
14	Fuori da Matrix	www.facebook.com/123944574364433
15	Graviola Italia	www.facebook.com/130541730433071
16	Signoraggio.it	www.facebook.com/278440415537619
17	Informare Per Resistere	www.facebook.com/101748583911
18	Sul Nuovo Ordine Mondiale	www.facebook.com/340262489362734
19	Avvistamenti e Contatti	www.facebook.com/352513104826417
20	Umani in Divenire	www.facebook.com/195235103879949
21	Nikola Tesla—il SEGRETO	www.facebook.com/108255081924
22	Teletrasporto	www.facebook.com/100774912863
23	PNL e Ipnosi	www.facebook.com/150500394993159
24	HAARP—controllo climatico	www.facebook.com/117166361628599
25	Sezione Aurea, Studio di Energia Vibrazionale	www.facebook.com/113640815379825
26	PER UNA NUOVA MEDICINA	www.facebook.com/113933508706361
27	PSICOALIMENTARSI E CURARSI NATURALMENTE	www.facebook.com/119866258041409
28	La nostra ignoranza la LORO forza.	www.facebook.com/520400687983468
29	HIV non causa AIDS	www.facebook.com/121365461259470
30	Sapere un Dovere	www.facebook.com/444729718909881
31	V per Verità	www.facebook.com/223425924337104
32	Genitori veg	www.facebook.com/211328765641743
33	Operatori di luce	www.facebook.com/195636673927835
34	Coscienza Nuova	www.facebook.com/292747470828855
35	Aprite Gli Occhi	www.facebook.com/145389958854351
36	Neovitruvian	www.facebook.com/128660840526907
37	CoscienzaSveglia	www.facebook.com/158362357555710
38	Medicinenon	www.facebook.com/248246118546060
39	TERRA REAL TIME	www.facebook.com/208776375809817
